# Quality of stroke guidelines in low- and middle-income countries: a systematic review

**DOI:** 10.2471/BLT.21.285845

**Published:** 2021-06-29

**Authors:** Joseph Yaria, Artyom Gil, Akintomiwa Makanjuola, Richard Oguntoye, J Jaime Miranda, Maria Lazo-Porras, Puhong Zhang, Xuanchen Tao, Jhon Álvarez Ahlgren, Antonio Bernabe-Ortiz, Miguel Moscoso-Porras, German Malaga, Irina Svyato, Morenike Osundina, Camila Gianella, Olamide Bello, Abisola Lawal, Ajagbe Temitope, Oluwadamilola Adebayo, Monkol Lakkhanaloet, Michael Brainin, Walter Johnson, Amanda G Thrift, Jurairat Phromjai, Annabel S Mueller-Stierlin, Sigiriya Aebischer Perone, Cherian Varghese, Valery Feigin, Mayowa O Owolabi, on behalf of the Stroke Experts Collaboration Group

**Affiliations:** a Department of Medicine, University College Hospital, Ibadan, Nigeria.; b Division of Country Health Programme, WHO European Office for the Prevention and Control of Noncommunicable Diseases, Moscow, Russia.; c School of Medicine, Universidad Peruana Cayetano Heredia, Lima, Peru.; d CRONICAS Centre of Excellence in Chronic Diseases, Universidad Peruana Cayetano Heredia, Lima, Peru.; e The George Institute for Global Health, Beijing, China.; f Department of Global Public Health, Karolinska Institutet, Solna, Sweden.; g Moscow School of Management SKOLKOVO, Moscow, Russia.; h Department of Psychology, Pontificia Universidad Católica del Perú, Lima, Peru.; i Thung Chang Hospital, Thung Chang District, Nan, Thailand.; j Department of Neurosciences and Preventive Medicine, Danube University, Krems, Austria.; k Department of Neurosurgery, Loma Linda University, California, United States of America.; l School of Clinical Sciences, Monash University, Melbourne, Australia.; m Health System Research Institute, Nonthaburi, Thailand.; n Institute for Epidemiology and Medical Biometry, University of Ulm, Ulm, Germany.; o Division of Tropical and Humanitarian Medicine, Geneva University Hospitals, Geneva, Switzerland.; p Noncommunicable Disease Department, World Health Organization, Geneva, Switzerland.; q National Institute for Stroke and Applied Neurosciences, Auckland University of Technology, Auckland, New Zealand.

## Abstract

**Objective:**

To identify gaps in national stroke guidelines that could be bridged to enhance the quality of stroke care services in low- and middle-income countries.

**Methods:**

We systematically searched medical databases and websites of medical societies and contacted international organizations. Country-specific guidelines on care and control of stroke in any language published from 2010 to 2020 were eligible for inclusion. We reviewed each included guideline for coverage of four key components of stroke services (surveillance, prevention, acute care and rehabilitation). We also assessed compliance with the eight Institute of Medicine standards for clinical practice guidelines, the ease of implementation of guidelines and plans for dissemination to target audiences.

**Findings:**

We reviewed 108 eligible guidelines from 47 countries, including four low-income, 24 middle-income and 19 high-income countries. Globally, fewer of the guidelines covered primary stroke prevention compared with other components of care, with none recommending surveillance. Guidelines on stroke in low- and middle-income countries fell short of the required standards for guideline development; breadth of target audience; coverage of the four components of stroke services; and adaptation to socioeconomic context. Fewer low- and middle-income country guidelines demonstrated transparency than those from high-income countries. Less than a quarter of guidelines encompassed detailed implementation plans and socioeconomic considerations.

**Conclusion:**

Guidelines on stroke in low- and middle-income countries need to be developed in conjunction with a wider category of health-care providers and stakeholders, with a full spectrum of translatable, context-appropriate interventions.

## Résumé

**Objectif:**

Identifier les lacunes présentes dans les directives nationales de prise en charge des accidents vasculaires cérébraux (AVC), ainsi que la manière de les combler afin d'améliorer la qualité des soins dans les pays à faible et moyen revenu.

**Méthodes:**

Nous avons analysé systématiquement une série de bases de données médicales et sites Internet de sociétés médicales. Nous avons également contacté des organismes internationaux. Toutes les directives nationales de prévention et de prise en charge des AVC, publiées dans n'importe quelle langue entre 2010 et 2020, étaient admises dans cette analyse. Nous avons examiné chaque directive incluse à la recherche de quatre composants clés de la prise en charge des AVC (surveillance, prévention, soins intensifs et rééducation). Enfin, nous avons évalué le degré de conformité avec les huit standards de l'Institut de médecine pour les directives de pratique clinique, mais aussi la facilité avec laquelle ces directives étaient appliquées et la manière dont elles étaient censées être diffusées auprès du public cible.

**Résultats:**

Nous avons étudié 108 directives retenues provenant de 47 pays dont quatre à faible revenu, 24 à moyen revenu et 19 à haut revenu. Globalement, le nombre de directives concernant la prévention primaire des AVC était moins élevé que d'autres volets de soins, et aucune ne recommandait une surveillance. Les directives relatives aux AVC dans les pays à faible et moyen revenu ne répondaient pas aux exigences en matière de développement des directives; d'étendue du public cible; d'intégration des quatre composants de prise en charge des AVC; ni d'adaptation au contexte socioéconomique. La transparence des directives nationales était moins souvent assurée dans les pays à faible et moyen revenu que dans les pays à haut revenu. Moins d'un quart des directives comportaient des plans de mise en œuvre détaillés et des considérations économiques.

**Conclusion:**

Les directives relatives à la prise en charge des AVC dans les pays à faible et moyen revenu doivent être renforcées avec le concours d'une plus large catégorie de prestataires et d'acteurs du secteur de la santé. Elles doivent en outre prévoir une gamme complète d'interventions transposables sur le terrain et adaptées au contexte.

## Resumen

**Objetivo:**

Identificar las lagunas en las directrices nacionales sobre accidentes cerebrovasculares que podrían subsanarse para mejorar la calidad de los servicios de atención a los accidentes cerebrovasculares en los países con ingresos bajos y medios.

**Métodos:**

Se realizaron búsquedas sistemáticas en bases de datos médicas y sitios web de sociedades médicas y se contactó con organizaciones internacionales. Se incluyeron las directrices específicas de cada país sobre la atención y el control de los accidentes cerebrovasculares en cualquier idioma, publicadas entre 2010 y 2020. Se revisó cada directriz incluida para la cobertura de cuatro componentes clave de los servicios por ataques cerebrovasculares (vigilancia, prevención, atención aguda y rehabilitación). También se evaluó el cumplimiento de los ocho estándares del Instituto de Medicina para las guías de práctica clínica, la facilidad de implementación de las directrices y los planes de difusión al público objetivo.

**Resultados:**

Se revisaron 108 directrices elegibles de 47 países, incluyendo cuatro de bajos ingresos, 24 de medianos ingresos y 19 de altos ingresos. Globalmente, la prevención primaria de los ataques cerebrovasculares estaba cubierta en menos directrices que otros componentes de la atención médica, y ninguna recomendaba la vigilancia. Las directrices sobre ataques cerebrovasculares en los países de ingresos bajos y medios no cumplían con los estándares requeridos para el desarrollo de directrices; la amplitud del público objetivo; la cobertura de los cuatro componentes de los servicios de ataques cerebrovasculares; ni la adaptación al contexto socioeconómico. El número de directrices de los países de ingresos bajos y medios que demostraron ser transparentes fue menor que el de los países de ingresos altos. Menos de una cuarta parte de las directrices incluían planes de aplicación detallados y consideraciones socioeconómicas.

**Conclusión:**

Las directrices sobre los ataques cerebrovasculares en los países de ingresos bajos y medios deben elaborarse en colaboración con una categoría más amplia de proveedores de servicios sanitarios y partes interesadas, con un espectro completo de intervenciones traducibles y adecuadas al contexto.

## ملخص

**الغرض:**

تحديد الفجوات في المبادئ التوجيهية الوطنية للسكتة الدماغية التي يمكن سدها لتحسين جودة خدمات رعاية السكتة الدماغية في البلدان منخفضة الدخل والبلدان متوسطة الدخل.

**الطريقة:**

قمنا بإجراء البحث بشكل منهجي في قواعد البيانات الطبية والمواقع الإلكترونية للجمعيات الطبية، واتصلنا بالمنظمات الدولية. إن المبادئ التوجيهية الخاصة بكل بلد حول رعاية السكتة الدماغية والسيطرة عليها، بأية لغة منشورة في الفترة من 2010 إلى 2020، كانت مؤهلة ليتم تضمينها. قمنا بمراجعة كل مبدأ توجيهي متضمن لتغطية أربعة مكونات رئيسية لخدمات السكتة الدماغية (المراقبة، والوقاية، والرعاية الدقيقة، وإعادة التأهيل). كما قمنا بتقييم الامتثال لمعايير معهد الطب الثمانية للمبادئ التوجيهية للممارسة الإكلينيكية، وسهولة تنفيذ المبادئ التوجيهية، وخطط النشر بين الجماهير المستهدفة.

**النتائج:**

قمنا بمراجعة 108 من المبادئ التوجيهية المؤهلة من 47 بلدًا، بما في ذلك أربعة بلدان منخفضة الدخل، و24 بلدًا متوسط الدخل، و19 بلدًا مرتفع الدخل. على الصعيد العالمي، غطى القليل من المبادئ التوجيهية الوقاية الأولية من السكتة الدماغية، مقارنةً بمكونات الرعاية الأخرى، مع عدم توصية أي منها بالمراقبة. كانت المبادئ التوجيهية حول السكتة الدماغية في البلدان منخفضة الدخل والبلدان متوسطة الدخل أقل من المعايير المطلوبة لتطوير المبادئ التوجيهية؛ وقاعدة الجمهور المستهدف؛ وتغطية المكونات الأربعة لخدمات السكتة الدماغية؛ والتكيف مع السياق الاجتماعي والاقتصادي. عدد المبادئ التوجيهية للبلدان منخفضة الدخل ومتوسطة الدخل التي أظهرت الشفافية أقل من المبادئ التوجيهية من البلدان ذات الدخل المرتفع. اشتمل أقل من ربع المبادئ التوجيهية على خطط تفصيلية للتنفيذ والاعتبارات الاجتماعية الاقتصادية.

**الاستنتاج:**

إن المبادئ التوجيهية حول السكتة الدماغية في البلدان ذات الدخل المنخفض، والبلدان ذات الدخل المتوسط، تحتاج إلى تطويرها بالاشتراك مع فئة أكبر من مقدمي الرعاية الصحية وأصحاب المصلحة، مع نطاق كامل من التدخلات القابلة للتفسير والملائمة للسياق.

## 摘要

**目的:**

旨在确定国家中风指南存在的差距，并对这些差距加以弥补以增强中低收入国家中风护理服务的质量。

**方法:**

我们系统性地检索了医学数据库和医学会网站，并联系了国际组织。自 2010 年至 2020 年以任何语言发表的针对特定国家的中风护理和控制指南均满足纳入资格。我们回顾了各个指南所涵盖的中风服务四大关键要素（监测、预防、紧急护理和康复）。我们还评估了医学研究所八项临床实践指南标准的遵守情况，指南实施的难易程度以及出于目标群体宣传目的而制定的计划。

**结果:**

我们回顾了来自 47 个国家（其中包括 4 个低收入国家、24 个中等收入国家和 19 个高收入国家）的 108 份符合条件的指南。整体而言，与其他护理要素相比，涵盖初级中风预防的指南较少，且没有指南针对监测给出建议。低收入和中等收入国家的中风指南在以下方面低于指南制定所需达到的标准：目标受众的广度、涵盖中风服务的四大要素以及对社会经济环境的适应程度。与高收入国家的指南相比，低收入和中等收入国家的指南透明度较低。不到四分之一的指南涵盖详细的实施计划和社会经济学方面的考虑因素。

**结论:**

低收入和中等收入国家的中风指南需要与更广泛的医疗护理提供者以及利益相关者共同制定，并因地制宜地采取一系列可转化的干预调控措施。

## Резюме

**Цель:**

Выявить пробелы в национальных рекомендациях по инсульту, которые возможно устранить с целью повышения качества услуг по лечению инсульта в странах с низким и средним уровнем доходов.

**Методы:**

Авторы систематически проводили поиск в медицинских базах данных и на веб-сайтах медицинских обществ, а также связывались с международными организациями. Национальные рекомендации по лечению и контролю инсульта, опубликованные с 2010 по 2020 г. на любом языке, соответствовали критериям для включения. Авторы изучили каждую включенную рекомендацию в отношении охвата четырьмя ключевыми компонентами услуг по инсульту (эпиднадзор, профилактика, неотложная помощь и реабилитация). Было также оценено соответствие восьми стандартам Института медицины для рекомендаций по клинической практике, простота внедрения рекомендаций и планы по их распространению среди целевых аудиторий.

**Результаты:**

Авторы изучили 108 подходящих рекомендаций из 47 стран, включая четыре страны с низким уровнем доходов, 24 страны со средним уровнем доходов и 19 стран с высоким уровнем доходов. Во всем мире первичная профилактика инсульта охватывала меньшее количество рекомендаций по сравнению с другими компонентами медицинской помощи, и ни один из них не рекомендовал эпиднадзор. Рекомендации по инсульту в странах с низким и средним уровнем доходов не соответствовали требуемым стандартам для разработки рекомендаций, широте целевой аудитории, охвату четырьмя компонентами услуг по инсульту и адаптации к социально-экономическим условиям. Рекомендации, принятые в странах с низким и средним уровнем доходов, в меньшей степени демонстрировали прозрачность, в отличие от рекомендаций для стран с высоким уровнем дохода. Менее четверти рекомендаций включали подробные планы по реализации и социально-экономические соображения.

**Вывод:**

Рекомендации по инсульту в странах с низким и средним уровнем доходов необходимо разрабатывать совместно с более широкой категорией поставщиков медицинских услуг и заинтересованных сторон, с полным спектром реализуемых и соответствующих обстановке вмешательств.

## Introduction

Stroke is the second leading cause of death and disability globally, with evidence of an increasing incidence of stroke among young adults.^1^^–^^3^ The burden of stroke is increasing in low- and middle-income countries.^4^ Studies have shown a 37% increase in the number of deaths among younger adults aged 20–64 years in low- and middle-income countries, from 942 921 to 1 292 347, versus a 20% decline in high-income countries over the period 1990–2013, from 236 566 to 191 359.^4^ Improvements in the prevention and management of stroke after implementation of evidence-based guidelines in routine medical practice have substantially lowered the incidence and mortality rates of stroke in high-income countries over the past 30 years.^1^^,^^3^^,^^5^^–^^8^ In contrast, low- and middle-income countries present wide differences in the quality of stroke prevention and care, with gaps identified in the knowledge and skills of health professionals, the resources available within health systems and the components of stroke care available locally.^6^^,^^9^ Addressing these gaps could be aided by guidelines with pragmatic evidence-based recommendations and implementation action plans for individuals and health systems.^10^ However, successful implementation of guidelines depends on having locally developed content in which region-specific barriers and local sociocultural characteristics are considered.^11^^–^^13^

We conducted a systematic review to compare recent clinical guidelines on stroke in low- and middle-income countries with those of high-income countries. We aimed to characterize specific gaps in guideline development, target audiences and content in relation to the spectrum of stroke care covered^14^ and the features that promote implementation. Our review was informed by the view that the content of guidelines for low- and middle-income countries should be adapted with solutions that are pragmatic for these countries and perhaps graded according to ease of implementation.^15^ Periodic review of published stroke guidelines is also important to improve their impact on stroke prevention and outcomes.

## Methods

We pre-registered the proposed methods for this systematic review on the International Prospective Register of Systematic Reviews (CRD42018112620). The review was conducted using the Preferred Reporting Items for Systematic Reviews and Meta-Analyses guidelines^16^ as well as procedures used by the Global Alliance for Chronic Diseases group for the systematic review of guidelines for hypertension and diabetes mellitus.^14^^,^^17^^,^^18^

### Search strategy

We searched the following electronic medical databases for published guidelines on management and prevention of stroke: PubMed®, African Journals Online, Directory of Open Access Journals, Google Scholar, SciELO and Excerpta Medica Database (EMBASE). We based our search strategy on the PICO strategy^19^ of evidence-based models (population: stroke guidelines; intervention: not applicable; comparison: guidelines from high-income countries versus those from low- and middle-income countries; outcome: spectrum of stroke care). We used medical subject headings and titles containing the following search terms: “country name” AND  “guideline” OR “consensus” OR “clinical protocols” OR “standards” OR “recommendations” AND  “stroke OR cerebrovascular disorder/disease OR intracranial haemorrhage OR cerebrovascular accident”. We also used the Google search engine to identify stroke guidelines published on the websites of medical societies. To identify additional guidelines, we contacted country representatives on the *Lancet Neurology* Commission on Stroke (listed in the authors’ data repository),^20^ members of the World Stroke Organization and the Global Alliance of Health Research Funders.^15^

Three of the authors independently screened the titles of records from the above-mentioned sources. Three authors independently reviewed the title, year of publication, publication type and author. This information was collated by one author and duplicates and irrelevant records based on the reviewers’ decisions were removed. Abstracts of each relevant title were independently reviewed for eligibility by three authors and the relevant publications were obtained for review. Additional publications obtained were screened by one author to determine their eligibility before inclusion. Reviewers with experience in stroke care in each participating country assisted in reviewing guidelines that were not published in English language. 

We included all country-specific stroke guidelines published from 2010 to 2020 regardless of the language. To avoid duplication, we selected the most recent guidelines where there were two or more guidelines. We excluded guidelines if they were designed exclusively for management of stroke in younger people (age < 45 years). However, we included guidelines concerning the management of stroke in the young among other age groups.

### Data extraction

Relevant data from each guideline were extracted independently by at least two researchers into a pre-designed structured evaluation form (available in the data repository).^20^ The extracted data were reassessed for consistency by a different reviewer and in the event of contradictory entries, the publications were cross-checked by an independent reviewer. Non-English language guidelines were reviewed independently by at least two researchers fluent in the language. In the event of differing opinions between reviewers, we carried out a joint review to arrive at a consensus.

The design of the proforma allowed us to assess each guideline’s coverage of the four key components of stroke services: (i) epidemiological surveillance; (ii) stroke prevention (primary and secondary); (iii) acute care; and (iv) rehabilitation.^14^ Primary and secondary stroke prevention and treatment covered stroke risk factors such as hypertension, smoking, diabetes, dyslipidaemia and atrial fibrillation. We assessed acute stroke care in the following categories: pre-hospital care; management of blood pressure, fever, glucose, oedema and seizures; ischaemic stroke care (including thrombolysis); intracerebral haemorrhage care; and subarachnoid haemorrhage care. Rehabilitation covered: dysphagia care; prophylaxis of deep venous thrombosis; depression care; education; physiotherapy nursing; and speech and cognition therapy.

We determined if a guideline was published by a stroke-related organization (such as a professional medical society) or government health ministry. Also, each guideline was assessed based on the Institute of Medicine eight quality standards for the development of trustworthy clinical practice guidelines: (i) transparency; (ii) management of conflict of interest; (iii) composition of guideline development group; (iv) use of systematic review; (v) grading rated by strength of recommendations; (vi) articulation of recommendations; (vii) external review; and (viii) proposed date for future review.^21^ We categorized the target audience for guidelines into health-care providers, patients, general population, policy-makers, payers (health-care funders) or implementation partners.^14^ We determined the guideline content by assessing which services were covered on the spectrum of stroke care and the characteristics that promote guideline implementation – contextualization (translatability); a clear implementation plan or dissemination plan; economic considerations; social considerations; legal considerations; and ethical considerations. A guideline was deemed to have considered ethical, legal, social and economic issues if it included information about ethical dilemmas, stroke-related legal issues, social issues and stroke financing. If the required information was not stated by the guidelines, we scored the guideline as not having addressed them. A guideline was deemed to be translatable if locally sourced interventions were stated or the recommendations were graded according to the resources required for implementation.

### Data analysis

We analysed the data collected using Stata statistical software, version 12 (StataCorp, College Station, United States of America). We report the frequencies and percentage of guidelines by country income group using the 2020 World Bank classification.^22^ We used the total number of countries or total number of guidelines as denominators.

## Results

**Fig. 1:**
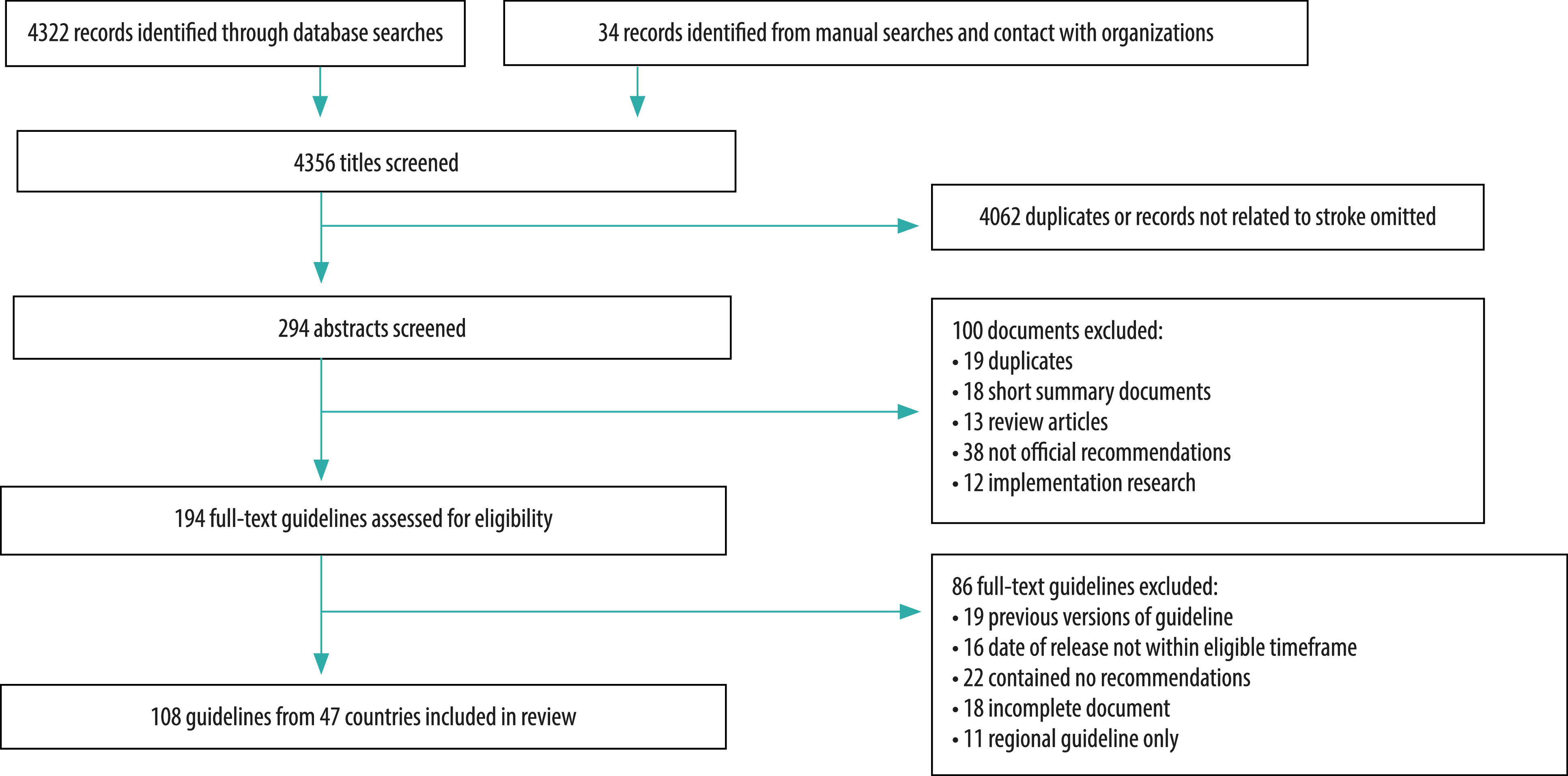
Flowchart of documents selected for the systematic review of guidelines on stroke care

After screening 4356 records from the literature search, we included 108 national guidelines from 47 countries in the final analysis (Fig. 1).^23^^–^^131^ We found four guidelines from 4 (14%) of the 29 World Bank low-income group countries (Ethiopia, Rwanda, Somalia, Uganda); 13 guidelines from 9 (18%) of 50 lower-middle-income countries (Cameroon, El Salvador, India, Kenya, Mongolia, Pakistan, Philippines, Solomon Islands and Sri Lanka); 24 guidelines from 15 (27%) of 56 upper-middle-income countries (Argentina, Brazil, China, Colombia, Ecuador, Georgia, Guatemala, Malaysia, Mexico, Namibia, Peru, Russian Federation, South Africa, Thailand and Tuvalu); and 67 guidelines from 19 (23%) of 83 high-income countries or territory (Australia, Canada, Chile, Germany, Ireland, Italy, Japan, Netherlands, New Zealand, Oman, Qatar, Republic of Korea, Singapore, Spain, Sweden, Switzerland, Taiwan, China, United Kingdom of Great Britain and Northern Ireland, and United States of America) (Table 1; available at https://www.who.int/publications/journals/bulletin/).

**Table 1:** Guidelines included in the systematic review of guidelines on stroke care

Author	Country	Income group	Year of study	Organization	Scope	Target audience
Bryer et al.^27^	South Africa	Upper-middle	2010	South African Stroke Society; National Department of Health	Primary prevention; secondary prevention; acute care; rehabilitation	Providers; public
Diener et al.^23^	Germany	High	2010	German Society of Neurology; German Stroke Society	Primary prevention; secondary prevention	NR
Irish Heart Foundation^24^	Ireland	High	2010	Irish Heart Foundation	Primary prevention; acute care; rehabilitation	Providers
Kamal et al.^26^	Pakistan	Lower-middle	2010	Pakistan Society of Neurology	Secondary prevention; acute care; rehabilitation	NR
Stroke Foundation of New Zealand^25^	New Zealand	High	2011	Stroke Foundation of New Zealand	Primary prevention; secondary prevention; acute care; rehabilitation	Providers; payers; policy-makers
Alonso de Leciñana et al.^36^	Spain	High	2011	Spanish Society of Neurology	Acute care	Providers
Bryer et al.^35^	South Africa	Upper-middle	2011	South African Stroke Society; National Department of Health	Acute care; rehabilitation	NR
Committee for Guidelines for Management of Aneurysmal Subarachnoid Hemorrhage^29^	Japan	High	2011	Japanese Society on Surgery for Cerebral Stroke	Acute care	Providers
Goldstein et al.^38^	United States	High	2011	American Heart Association; American Stroke Association	Primary prevention	Providers
Ministry of Health and Medical Services of Solomon Islands^34^	Solomon Islands	Lower-middle	2011	Ministry of Health and Medical Services of Solomon Islands	Secondary prevention	Providers
Ministry of Health and Social Services of Namibia^31^	Namibia	Upper-middle	2011	Ministry of Health and Social Services of Namibia	Acute care	NR
Quinn et al.^37^	United Kingdom	High	2011	Leeds General Infirmary	Acute care	NR
Staykov et al.^30^	Germany	High	2011	NR	Acute care	NR
Stroke Society of Philippines^32^	Philippines	Lower-middle	2011	Stroke Society of Philippines	Primary prevention; secondary prevention; acute care; rehabilitation	NR
Tsiskaridze^28^	Georgia	Upper-middle	2011	Ministry of Health of Georgia	Acute care	Providers
Venketasubramanian et al.^33^	Singapore	High	2011	Ministry of Health of Singapore	Secondary prevention; acute care; rehabilitation	Providers
Atallah^39^	Argentina	Upper-middle	2012	Stroke Council; Argentine Society of Cardiology	Acute care	Providers
Gonzalo et al.^44^	Ecuador	Upper-middle	2012	Ministry of Health of Ecuador	Secondary prevention	NR
Lansberg et al.^50^	United States	High	2012	American College of Chest Physicians	Acute care	Providers
Minematsu et al.^45^	Japan	High	2012	Japan Stroke Society	Acute care	Providers
Ministry of Health of Malaysia^47^	Malaysia	Upper-middle	2012	Malaysian Society of Neurosciences; Academy of Medicine Malaysia; Ministry of Health of Malaysia	Primary prevention; acute care	Providers
Ministry of Health of Mongolia^46^	Mongolia	Lower-middle	2012	Ministry of Health of Mongolia	Primary prevention; secondary prevention; acute care; rehabilitation	Providers
National Drug and Therapeutic Committee^49^	Tuvalu	Upper-middle	2012	Ministry of Health of Tuvalu	Primary prevention; acute care	NR
National Vascular Disease Prevention Alliance^41^	Australia	High	2012	Royal Australian College of General Practitioners; National Vascular Disease Prevention Alliance	Primary prevention	Providers; policy-makers
Oliveira-Filho et al.^42^ and Martins et al.^43^^,a^	Brazil	Upper-middle	2012	Brazilian Stroke Society; Brazilian Academy of Neurology	Acute care	Providers
Vivancos et al.^48^	Spain	High	2012	Spanish Society of Neurology	Acute care	Providers
Alonso de Leciñana et al.^59^	Spain	High	2013	Spanish Society of Neurology	Acute care	Providers
Guatemalan Institute of Social Security^113^	Guatemala	Upper-middle	2013	Guatemalan Institute of Social Security	Rehabilitation	Providers
Lanza et al.^55^	Italy	High	2013	Italian Stroke Organization	Acute care	Providers
Liu et al.^53^	China	Upper-middle	2013	Expert Consensus group on the Evaluation and Intervention of Collateral Circulation for Ischaemic Stroke	Acute care	Providers
Ministry of Health of Mongolia^56^	Mongolia	Lower-middle	2013	Ministry of Health of Mongolia	Rehabilitation	Providers
Ministry of Health of Singapore^58^	Singapore	High	2013	Ministry of Health of Singapore; Academy of Medicine; College of Family Physicians; Clinical Neuroscience Society; Singapore National Stroke Association; College of Physicians	Acute care	Providers
Ministry of Health of Thailand^60^	Thailand	Upper-middle	2013	Royal College of Surgeons of Thailand; Royal College of Physicians of Thailand; Royal College of Rehabilitation Medicine of Thailand; Neuroscience Society of Thailand; College of Neurosurgeons of Thailand; Thai Stroke Association; Office of Medical Academic Development; Ministry of Health of Thailand	Acute care; rehabilitation	Providers
National Institute for Health and Care Excellence^61^	United Kingdom	High	2013	National Institute for Health and Care Excellence	Rehabilitation	Providers; policy-makers; patients
North-West Region Best Practices in Stroke Rehabilitation Group^52^	Cameroon	Lower-middle	2013	North-West Region Best Practices in Stroke Rehabilitation Group; Bamenda Coordinating Centre for Studies in Disability and Rehabilitation; University of Toronto International Centre for Disability and Rehabilitation	Rehabilitation	Providers; patients; general population
Rivas et al.^54^	Chile	High	2013	Ministry of Health of Chile	Secondary prevention; acute care; rehabilitation	Providers
Steultjens et al.^57^	Netherlands	High	2013	Occupational Therapy Netherlands	Rehabilitation	Providers
Stroke Foundation of Australia^51^	Australia	High	2013	Stroke Foundation of Australia	Rehabilitation	Payers; policy-makers
Wintermark et al.^62^	United States	High	2013	American Society of Neuroradiology; American College of Radiology; Society of Neurointerventional Surgery	Acute care	Providers
Bushnell et al.^67^	United States	High	2014	American Heart Association; American Stroke Association	Primary prevention	Providers
Clinical Centre for Research in Aphasia Rehabilitation^40^	Australia	High	2014	Clinical Centre for Research in Aphasia Rehabilitation	Rehabilitation	NS
Hookway et al.^65^	United Kingdom	High	2014	British Diabetic Association; Royal College of Physicians	Secondary prevention	NR
Kernan et al.^66^	United States	High	2014	American Heart Association; American Stroke Association	Secondary prevention	Providers
Wang et al.^64^	China	Upper-middle	2014	Chinese Society of Neurology; Cerebrovascular Disease Group	Secondary prevention	Providers
Wright et al.^63^	Australia	High	2014	National Stroke Foundation	Secondary prevention; acute care; rehabilitation	Providers
All-Russian Society of Neurologists^84^	Russian Federation	Upper-middle	2015	All-Russian Society of Neurologists; Association of Neurosurgeons of the Russian Federation; Association of Neuro-Anesthesiologists and Neuro-Resuscitators; Union of Rehabilitologists of the Russian Federation	Primary prevention; secondary prevention; acute care; rehabilitation	NR
Berns et al.^83^	Netherlands	High	2015	Dutch Association of Aphasia Therapists; Dutch Association for Speech Therapy and Phoniatrics	Rehabilitation	Providers; payers
Bösel et al.^75^	Germany	High	2015	German Society for Neurology; Neurocritical Care Society	Acute care	Providers
Casaubon et al.^72^	Canada	High	2015	Heart and Stroke Foundation of Canada	Acute care	Providers
Clinical Research Centre for Stroke^86^	Republic of Korea	High	2015	Clinical Research Centre for Stroke	Primary prevention; secondary prevention; acute care	Providers
Dalal et al.^81^	India	Lower-middle	2015	Stroke Prevention in Atrial Fibrillation Academy India Experts	Secondary prevention	NR
Eskes et al.^70^	Canada	High	2015	Heart and Stroke Foundation of Canada	Rehabilitation	Providers
Gunaratne et al.^87^	Sri Lanka	Lower-middle	2015	Ministry of Health of Sri Lanka	Secondary prevention; acute care; rehabilitation	Providers
Harris et al.^69^	Canada	High	2015	Canadian Association of Emergency Physicians	Acute care	Providers
Hebert et al.^71^	Canada	High	2015	Heart and Stroke Foundation of Canada	Rehabilitation	Providers
Hemphill et al.^88^	United States	High	2015	American Heart Association	Acute care	Providers
Koziolek & Lüders^77^	Germany	High	2015	NR	Acute care	NR
McTaggart et al.^73^	Canada	High	2015	Society of Neuro-Interventional Surgery	Acute care	Providers
Ministry of Health of Argentina^68^	Argentina	Upper-middle	2015	National Disease Prevention and Control Program; Cardiovascular Directorate of Health Promotion and Disease Control; Ministry of Health of Argentina	Acute care	Providers; policy-makers
Möhlenbruch & Bendszus^79^	Germany	High	2015	NR	Acute care	NR
Nabavi et al.^78^	Germany	High	2015	German Stroke Society. German Stroke Foundation	Acute care	Providers
Nolte & Audebert^76^	Germany	High	2015	NR	Acute care	NR
Somali Health Authorities^85^	Somalia	Low	2015	Somali Health Authorities; World Health Organization	Secondary prevention	Providers
Toni et al.^82^	Italy	High	2015	Italian Stroke Organization	Acute care	Providers
Torbey et al.^80^	Germany	High	2015	Neurocritical Care Society and German Society for Neuro-Intensive Care and Emergency Medicine	Acute care	Providers
Turriago et al.^74^	Colombia	Upper-middle	2015	Ministry of Health of Columbia	Acute care; rehabilitation	Providers
Cameron et al.^90^	Canada	High	2016	Heart and Stroke Foundation of Canada	Rehabilitation	Providers; patients; general population
Casaubon et al.^91^	Canada	High	2016	Heart and Stroke Foundation of Canada	Acute care	Providers
Gebremichael et al.^92^	Ethiopia	Low	2016	Ministry of Health of Ethiopia	Acute care	Providers
Glober et al.^99^	United States	High	2016	Emergency Medical Services; Medical Directors Association of California	Acute care	Providers
Jung et al.^131^	Switzerland	High	2016	Bern Stroke Centre	Secondary prevention; acute care	NR
Kim et al.^93^	Republic of Korea	High	2016	Korea Society for Neurorehabilitation	Rehabilitation	Providers
Ministry of Health of Rwanda^95^	Rwanda	Low	2016	Ministry of Health of Rwanda	Acute care; rehabilitation	Providers
Ministry of Health of Uganda^97^	Uganda	Low	2016	Ministry of Health of Uganda	Acute care	Providers
Ministry of Public Health of Qatar^94^	Qatar	High	2016	Ministry of Public Health of Qatar	Secondary prevention; acute care; rehabilitation	Providers
Pontes-Neto et al.^89^	Brazil	Upper-middle	2016	Brazilian Stroke Society; Brazilian Academy of Neurology; Brazilian Stroke Network; Brazilian Society of Diagnostic and Therapeutic Neuroradiology	Acute care	Providers; policy-makers
Taiwan Stroke Society^96^	Taiwan, China	High	2016	Taiwan Stroke Society	Primary prevention; secondary prevention; acute care; rehabilitation	Providers
Winstein et al.^98^	United States	High	2016	American Heart Association; American Stroke Association	Rehabilitation	Providers
Bertoluci et al.^100^	Brazil	Upper-middle	2017	Brazilian Diabetes Society; Brazilian Society of Cardiology; Brazilian Endocrinology and Metabolism Society	Primary prevention	Providers
Dong et al.^102^	China	Upper-middle	2017	Chinese Stroke Association	Acute care	Providers
Guatemalan Institute of Social Security^104^	Guatemala	Upper-middle	2017	Guatemalan Institute of Social Security	Primary prevention; acute care	NR
Hong^106^	Republic of Korea	High	2017	Korean Stroke Society	Acute care	NR
Lanza et al.^105^	Italy	High	2017	Italian Stroke Organization	Acute care	Providers
Mexican Institute of Social Security^107^	Mexico	Upper-middle	2017	Directorate of Medical Benefits; Medical Care Unit; High Specialty Doctors; Technical Coordination of Clinical Excellence; Mexican Institute of Social Security	Acute care	NR
Ministry of Health of Chile^103^	Chile	High	2017	Public Health Disease Prevention and Control Division; Health Planning Division, Ministry of Health of Chile	Acute care	Providers
Ministry of Public Health of Qatar^111^	Qatar	High	2017	Ministry of Public Health of Qatar	Acute care; rehabilitation	Providers
Philippine Academy of Rehabilitation Medicine^110^	Philippines	Lower-middle	2017	Philippine Academy of Rehabilitation Medicine	Rehabilitation	Providers
Royal Dutch Society for Physiotherapy^108^	Netherlands	High	2017	Royal Dutch Society for Physical Therapy	Rehabilitation	Providers; patients
Royal Dutch Society for Neurology^109^	Netherlands	High	2017	Royal Dutch Society of Neurology	Secondary prevention; acute care; rehabilitation	Providers
Rudd et al.^112^	United Kingdom	High	2017	Royal College of Physicians	Secondary prevention; acute care; rehabilitation	Providers
Wein et al.^101^	Canada	High	2017	Heart and Stroke Foundation of Canada	Secondary prevention	Providers
Burkule et al.^116^	India	Lower-middle	2018	The Indian Academy of Echocardiography	Secondary prevention	NR
Escalante et al.^115^	El Salvador	Lower-middle	2018	Ministry of Health of El Salvador	Acute care	NR
Lee et al.^118^	Republic of Korea	High	2018	Korean Arrhythmia Society	Secondary prevention	NR
Ministry of Health of Kenya^117^	Kenya	Lower-middle	2018	Ministry of Health of Kenya	Primary prevention; secondary prevention; acute care	Providers
National Board of Health and Welfare of Sweden^120^	Sweden	High	2018	National Board of Health and Welfare of Sweden	Primary prevention; secondary prevention; acute care; rehabilitation	Providers; policy-makers
National Board of Health and Welfare of Sweden^121^	Sweden	High	2018	National Board of Health and Welfare of Sweden	Secondary prevention	Providers; policy-makers
NHG Working Group on Stroke^119^	Netherlands	High	2018	Dutch College of General Practitioners	Secondary prevention; acute care; rehabilitation	Providers
Zhao et al.^114^	China	Upper-middle	2018	Jiangsu Provincial Special Program of Medical Science	Rehabilitation	Providers
Dong et al.^123^	China	Upper-middle	2019	Chinese Stroke Association	Acute care	NR
Ko et al.^125^	Republic of Korea	High	2019	Korean Stroke Society	Acute care	NR
Ministry of Health and Family Welfare of India^124^	India	Lower-middle	2019	National Programme for Prevention and Control of Cancer, Diabetes, Cardiovascular Diseases and Stroke; Ministry of Health and Family Welfare of India	Primary prevention; secondary prevention; acute care; rehabilitation	Providers; policy-makers
National Institute for Health and Care Excellence^126^	United Kingdom	High	2019	National Institute for Health and Care Excellence	Acute care; rehabilitation	Providers; patients; policy-makers
Powers et al.^127^	United States	High	2019	American Heart Association; American Stroke Association	Acute care	Providers
Stroke Foundation of Australia^122^	Australia	High	2019	Stroke Foundation of Australia	Secondary prevention; acute care; rehabilitation	Providers; payers; policy-makers
Hornby et al.^130^	United States	High	2020	Academy of Neurologic Physical Therapy	Rehabilitation	NR
Ministry of Health of Oman^128^	Oman	High	2020	Ministry of Health of Oman	Acute care; rehabilitation	NR
Sequeiros-Chirinos et al.^129^	Peru	Upper-middle	2020	Peruvian Social Security Programme	Secondary prevention; acute care; rehabilitation	Providers

NR: not reported.

^a^ Oliveira-Filho et al.^42^ and Martins et al.^43^ are parts 1 and 2 of the same guideline.

### Guideline development

Of the included guidelines, 72 (67%) were published by stroke-related organizations, 25 (23%) by government health ministries and 7 (6%) by both stroke-related organizations and health ministries. The publisher was not specified for four guidelines. A higher proportion of the guidelines from high-income countries (54 out of 67; 81%) were published by a stroke-related organization than were guidelines from countries in other income groups (18 of 41; 44%). 

**Fig. 2:**
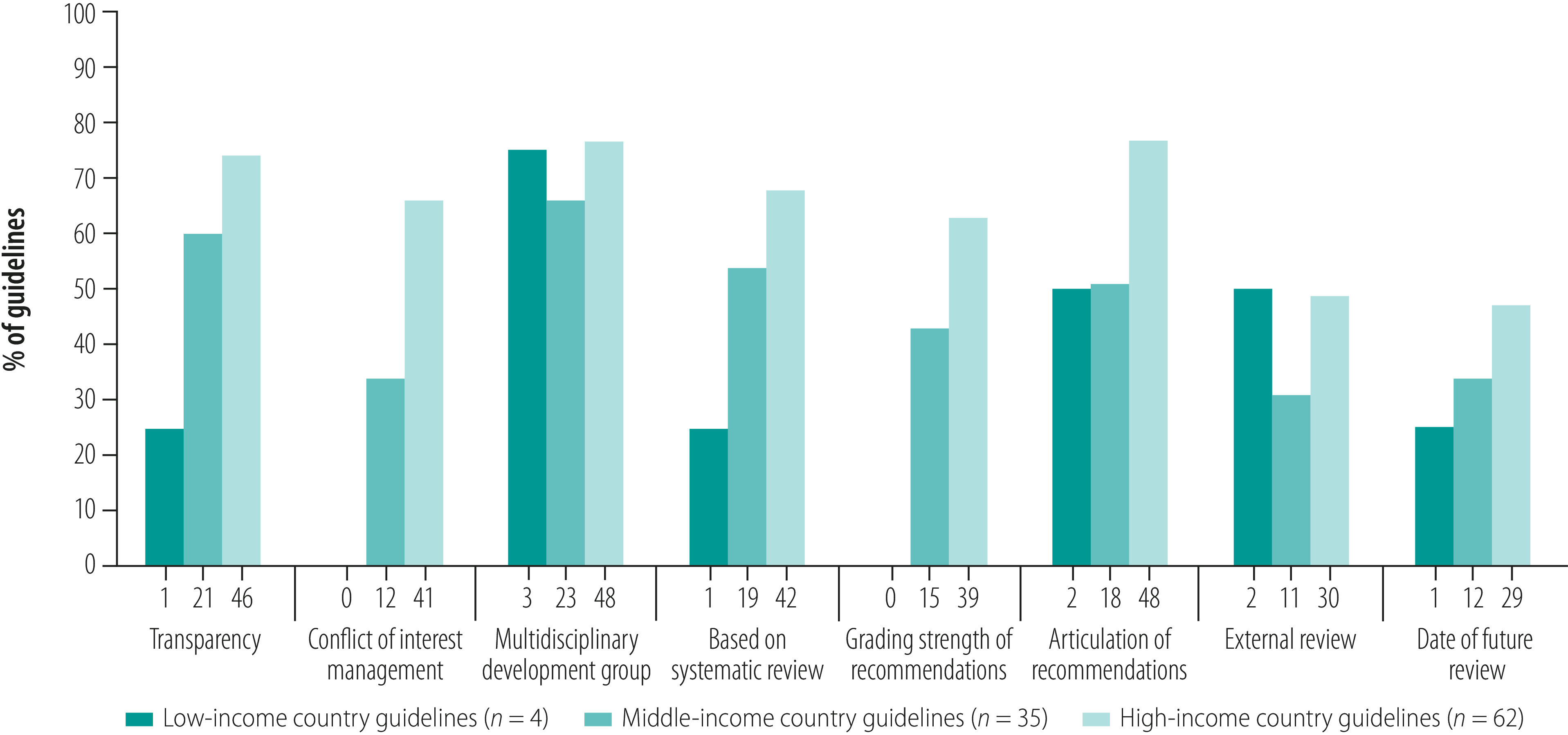
Proportion of stroke care guidelines satisfying the eight Institute of Medicine standards on guideline development

Notes: Country income groups are World Bank classifications.^22^ Categories are based on the Institute of Medicine standards for trustworthy clinical practice guidelines.^21^ The missing guidelines are those where there is no clear methods section.

Fig. 2 shows the profile of the included guidelines based on the eight Institute of Medicine standards. Just one (25%) of the low-income country guidelines established transparency in guideline development compared with 21 (60%) of the guidelines from middle-income countries and 46 (74%) of the guidelines from high-income countries. Similarly, one (25%) low-income country guideline was based on systematic reviews compared with 19 (54%) guidelines from middle-income countries and 42 (68%) guidelines from high-income countries. None of the low-income country guidelines graded the strength of their recommendations. 

### Target audience

Of the 81 guidelines that stated their target audience, all but one were directed towards health-care providers. None of the low- or middle-income country guidelines and four (8%) of the high-income country guidelines were directed at payers (health-care funders). Three (11%) of the middle-income country and eight (15%) of high-income country guidelines were directed at policy-makers. One (4%) guideline from middle-income countries and four (8%) from high-income countries targeted patients. Two (8%) of the middle-income country guidelines and one (2%) of the high-income country guidelines were targeted at the general population.

### Guideline content

**Fig. 3:**
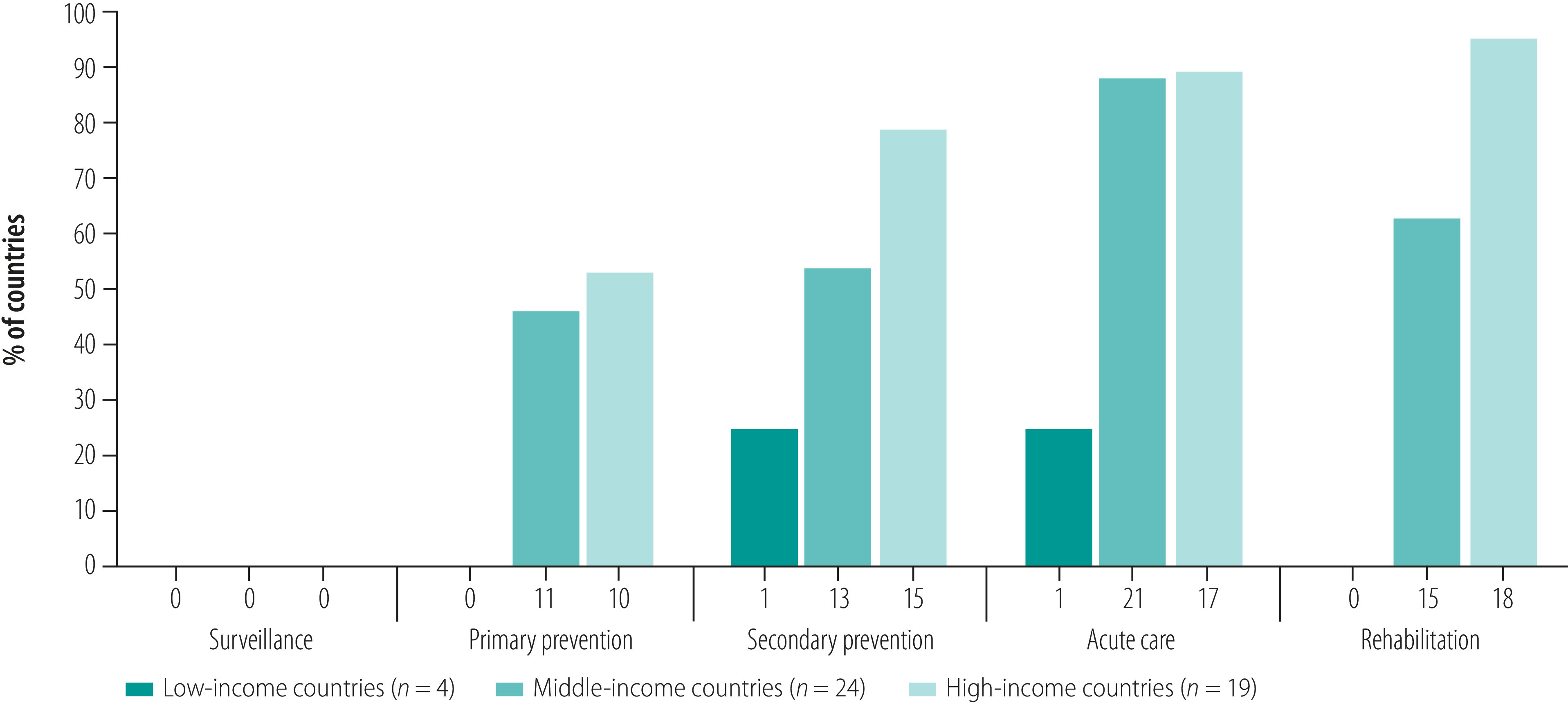
Proportion of countries with guidelines covering various components of stroke care

Note: Country income groups are World Bank classifications.^22^

On the spectrum of stroke interventions covered in each country (Fig. 3), we found 19 (40%) out of 47 countries had guidelines that covered primary prevention, 27 (57%) had guidelines addressing secondary prevention, 43 (91%) had guidelines covering acute care and 28 (60%) had guidelines addressing stroke rehabilitation. Of the guidelines assessed, a few documented stroke epidemiology in their various locales, but none specifically recommended epidemiological surveillance.

**Fig. 4:**
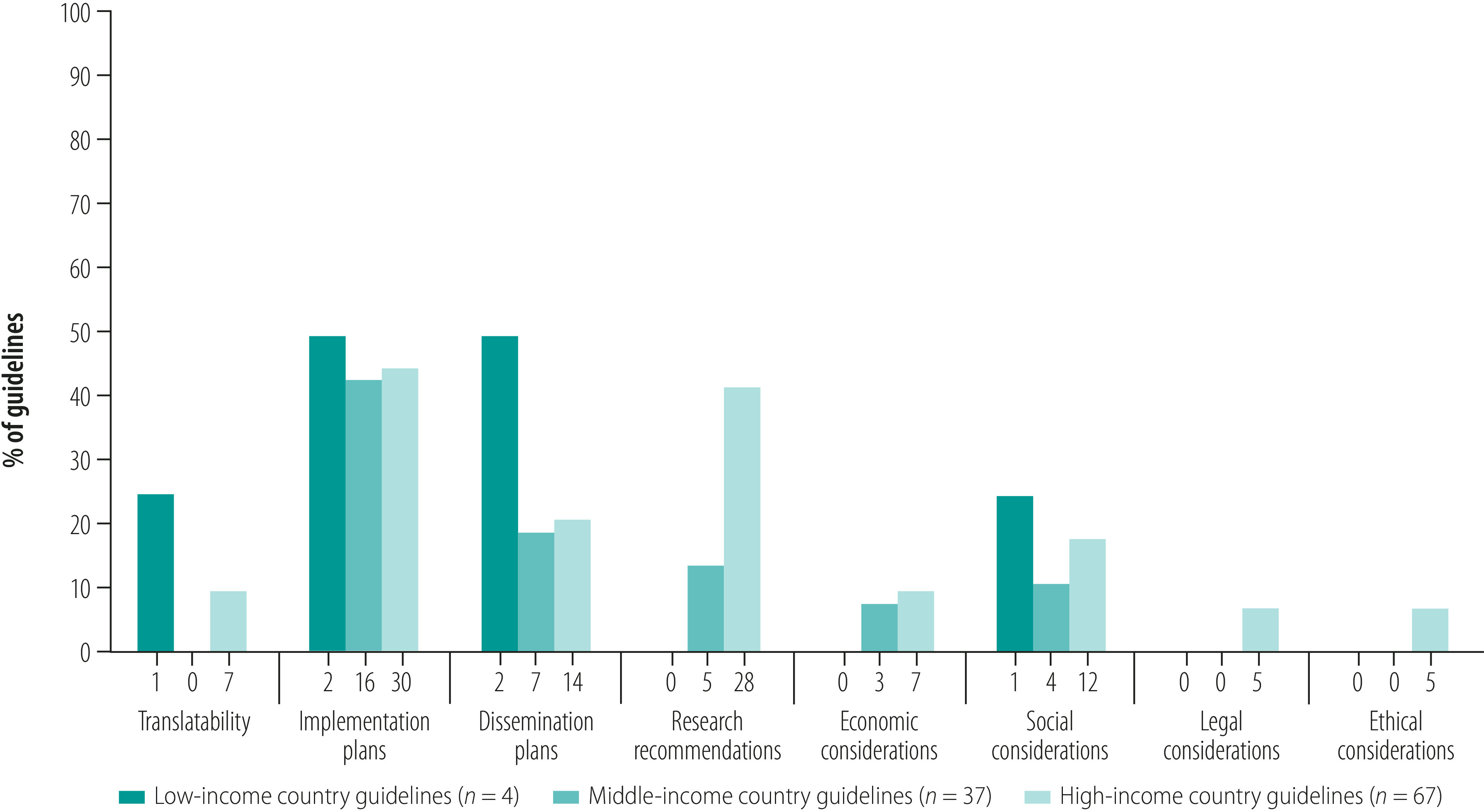
Proportion of stroke care guidelines with characteristics that promote implementation of guidelines

Notes: Country income groups are World Bank classifications.^22^

Only one (25%) of the low-income countries had a guideline that covered secondary stroke prevention in detail, while none dealt with diagnoses of cardiovascular risks or the use of anti-platelet therapy in detail (Fig. 3; see further details in the data repository).^20^ Globally, few guidelines considered implementation during the development process. One (25%) of the guidelines from low-income countries and seven guidelines (10%) from high-income countries ordered their recommendations by ease of implementation or gave locally sourced alternatives (Fig. 4). Similarly, economic implications were considered in seven (10%) and three (8%) guidelines from high-income countries and middle-income countries, respectively. Twenty-eight (42%) high-income country guidelines and five (14%) middle-income country guidelines gave research recommendations. 

## Discussion

Our study showed that national stroke guidelines from low- and middle-income countries, especially those from low-income countries, fell short in terms of quality, coverage and content. The implementation of recommended interventions in these countries may be hampered by factors such as shortages of health-care providers,^132^ limited access to health care,^133^ deficient infrastructure and ineffective health policies. Poor transportation and infrastructure and shortages of skilled personnel are the main factors responsible for suboptimal or unavailable pre-hospital stroke care.^134^ Acute stroke care is also affected by numerous factors including financial constraints, inadequate facilities and sociocultural practices.^135^ The health promotion strategies required for improved stroke prevention and stroke rehabilitation are also hampered by limited finances and lack of required resources. These constraints – rarely considered in the development of stroke guidelines for low- and middle-income countries – need to be addressed with pragmatic recommendations.

Previous studies have evaluated stroke guidelines, but have rarely investigated country-specific guidelines with regards to their development and ease of implementation in various settings. Each low- and middle-income country may need to analyse the capacity of its health system and identify weaknesses and barriers to the implementation of stroke guidelines. Such information is key to developing guidelines that would be relevant to the country context and hence more effective. Based on this information, recommendations should then be graded according to ease of implementation,^14^ with clear dissemination and implementation plans adapted to the country’s health system. We aimed to address these issues and offer pragmatic solutions for low- and middle-income countries.

In Latin America, countries such as Colombia and Mexico have made efforts towards building capacity for developing clinical guidelines to improve guidelines implementation. State agencies were involved in the development of national clinical guidelines, with open-access resources explaining the methods for the development of guidelines.^136^ These types of initiatives and resources could also assist in developing a translatability index for prioritizing or grading recommendations according to ease, cost and simplicity of implementation.^14^ However, funding is needed for guideline development and implementation. Availability of funds may explain the higher frequency of guidelines published by funded stroke-related organizations among high-income countries. Increasing the target audience for stroke guidelines to include policy-makers, health payers and implementation partners should stimulate collaboration in financing and sustaining pragmatic interventions.

Crucially, low- and middle-income countries should stop regarding guidelines as a tool solely for complex care at the hospital level, a bias suggested by our results. Guidelines should be designed as not only a tool for primary and specialized clinical care, but also as a guide for health planning and implementation, to enable better resource allocation and increased efficiency in stroke prevention and treatment. Expanding the target audience in future guidelines to include policy-makers, health payers and implementation partners is therefore an important step as most interventions require funding, policy initiatives and population buy-in. As shown in our review, none of the low- or middle-income country guidelines targeted payers, policy-makers, patients or the general population.

Pragmatic solutions in low- and middle-income countries require a wider reach of stroke guidelines through task-sharing, including the services of community health extension workers. A structured guideline-based programme involving health extension workers and other allied health professionals, possibly with supervision from stroke physicians, is worth exploring for rural communities and areas where health facilities are poorer quality or harder to access.^137^ Therefore, stroke guidelines could include clear instructions for immediate recognition of symptoms – who is responsible for care, what is to be done, when action or intervention should be taken, how this intervention is to be done and assessed, and a standard to guide referral practices. Simple measures to identify early stroke complications, prevention of stroke complications and necessary treatment (such as the Glasgow Coma Scale, the National Early Warning Score or limb girth), should be included in low- and middle-income country guidelines. Similarly, the resources and skills required at each hospital level can be stated, as listed for example in the guideline from Mongolia.^46^ This pragmatic approach is important, to improve implementation of guidelines from low- and middle-income countries towards addressing acute care, both for basic interventions and more advanced care. Reperfusion therapy, for example, is an effective intervention with cost–effectiveness analysis of more than 100 international dollars per disability-adjusted life years averted in low- and middle-income countries.^138^ Similarly, guideline recommendations need to reflect the sociocultural characteristics of each country, as cultural perspectives on diseases and care-seeking behaviour differ among countries.^139^

Notably, we found that stroke guidelines in low- and middle-income countries were not only deficient in quality but also in the spectrum of stroke prevention and care covered. None of the guidelines recommended stroke surveillance, a crucial component for monitoring, planning and evaluation of stroke burden and interventions.^140^ Primary and secondary stroke prevention also required improvement.^141^ For example, only one of the low-income countries had guidelines that covered secondary stroke prevention, while none had an independent stroke guideline that dealt with diagnoses of cardiovascular risks or the use of anti-platelet agents for secondary stroke prevention. The need for low- and middle-income countries to focus on stroke prevention is further strengthened by the success of high-income countries that has been rooted in primary and secondary prevention.^3^ In contrast, stroke guidelines from low- and middle-income countries had inadequate or no information on stroke prevention. A few of the low- and middle-income countries guidelines addressed major stroke risk factors, such as hypertension and diabetes, as well as feasible and effective population-wide strategies for primary stroke prevention. Nevertheless, these guidelines fell short of Institute of Medicine standards for trustworthiness and showed implementation gaps.^17^^,^^18^

In post-stroke care, where standard rehabilitation services may be lacking, stroke guidelines could indicate procedures for implementation of home-based or community-based rehabilitation care. Rehabilitation, nursing care, speech therapy and post-stroke cognition were not addressed in any of the low-income country guidelines, and less often in middle- than high-income country guidelines. In addition, including instructions for managing the unmet needs of caregivers who bear most of the burden of post-stroke care in low- and middle-income countries is needed.^142^ A comprehensive guideline-based programme with supervision is worth exploring for rural communities and areas with poor health facilities or access to care.^137^ Recommendations for community- or family-based rehabilitation, and the appropriate time to start them in the trajectory of stroke care, should be further explored as pragmatic interventions^143^ both in low- and middle-income countries and rural settings of high-income countries.^144^^,^^145^

Stroke care in low- and middle-income countries presents both challenges and opportunities for improvement. Guidelines in these countries may be more effective if properly adapted to the local context and disseminated for implementation by all stakeholders.^14^ It is important to address all the steps in the implementation cycle of guidelines for stroke care which includes content development, contextualization, dissemination to all stakeholders and evaluation.^14^ In countries that suffer from poor implementation of policies, addition of necessary details into national stroke guidelines may be a way of bringing the information directly to health-care providers and the general public. These cost-effective interventions can easily be adapted from already proven policy-related publications such as the World Health Organization recommended “best buys”,^138^ the health interventions for universal health coverage^146^ and other cost-effective interventions.

This review is not without its limitations as guidelines published online stood a higher chance of being included in the review. Also, guidelines available online but not published on any of the databases searched were unlikely to be included in the review as not every national association or official body could be individually contacted. However, to reduce this bias, we contacted stroke experts to determine the availability of additional guidelines that were not available online. Also, involving the World Health Organization more in the review process might have helped us to obtain more guidelines. 

In conclusion, the quality and implementation strategies of stroke guidelines need to be improved and adapted to the health-system context in low- and middle-income countries. To achieve this, the governments of these countries need to develop new guidelines or adapt existing guidelines in conjunction with a wider range of health-care providers and stakeholders. The intended target audience for stroke guidelines should be expanded to encourage effective communication with and commitment of all stakeholders. A full spectrum of translatable, context-appropriate interventions for stroke prevention, care and surveillance could deliver guidelines that are easier to implement and more effective.
